# Negative emotions concentrate while positive emotions diffuse in football fan rivalries

**DOI:** 10.1371/journal.pone.0356757

**Published:** 2026-09-24

**Authors:** Thomas U. Grund

**Affiliations:** Institute of Sociology, RWTH Aachen University, Aachen, Germany; University of Salerno, ITALY

## Abstract

**Background:**

Research on negativity bias demonstrates that negative information receives disproportionate cognitive attention at the individual level, but how this psychological mechanism shapes collective emotional patterns remains unclear. We investigate whether communities systematically concentrate negative emotions on fewer targets while distributing positive emotions more broadly.

**Methods:**

We analyze 36,223 German football fans’ reports of loved and hated clubs, measuring attention concentration using the Herfindahl-Hirschman Index (HHI). We distinguish between outgoing concentration (how communities allocate their own attention) and incoming concentration (how attention from others focuses on particular targets), testing whether concentration differs between positive and negative affect.

**Results:**

Negative attention was significantly more concentrated than positive attention for both outgoing (M = 0.36 vs M = 0.23, p < 0.001) and incoming ties (M = 0.41 vs M = 0.33, p = 0.011). Love and hate concentration are weakly correlated (r = 0.26–0.32), indicating independent processes. Structural factors predict incoming but not outgoing concentration (Pseudo *R*^2^ = 0.14–0.35 vs *R*^2^ = 0.47–0.49), revealing asymmetric mechanisms of attention allocation versus attraction.

**Conclusions:**

Communities face cognitive constraints that produce systematic asymmetries in how they distribute emotional attention. These findings highlight how cognitive constraints shape collective emotional structures, with implications for political polarization, consumer behavior, and conflict dynamics.

## Introduction

Negative information captures more attention and receives deeper cognitive processing than equivalent positive information—a phenomenon known as negativity bias [1,2]. This asymmetry operates powerfully at the individual level: negative stimuli automatically capture attention [3], receive more elaborate encoding [4], and generate stronger, longer-lasting emotional responses [5]. While negativity bias is well-documented at the individual level, its collective implications remain unclear.

This gap matters because communities regularly face attention allocation problems where they must distribute scarce emotional resources across multiple potential targets. Political movements must decide which opponents to focus their opposition against [6]. Consumer communities must allocate affection and animosity across competing brands [7]. Social groups must determine which outgroups merit sustained negative attention versus which can be safely ignored. If individual-level negativity bias aggregates to collective attention patterns, communities should systematically concentrate negative emotions on fewer targets while distributing positive emotions more broadly. This would create fundamentally different structures of conflict versus cooperation.

Existing research on positive and negative social ties primarily asks which actors form such relationships and what consequences those relationships have. It does not address the distributional question central here: when communities can direct emotional attention towards many possible targets, is negative attention systematically more concentrated than positive attention? Addressing this question requires comparing the distribution of positive and negative attention across a common set of potential targets rather than considering individual dyads in isolation.

Contemporary examples suggest such asymmetries may be widespread. Across politics, markets, and digital communication, negative attention increasingly concentrates on specific targets while positive identification distributes more broadly. Political polarization increasingly concentrates on specific opposing figures while positive identification distributes across multiple politicians and issues [8]. Brand hatred focuses intensely on particular competitors while brand appreciation spreads across numerous alternatives [7]. Online discourse exhibits concentrated negative attention on specific targets while positive engagement remains diffuse [9,10]. These patterns suggest that attention concentration asymmetries may reflect general principles of cognitive resource allocation rather than domain-specific dynamics.

We examine this question using data from German professional football (soccer), where 36,223 fans reported loved and hated clubs. Football provides an ideal empirical setting for several reasons. First, the multiplicity of available targets (79 clubs) creates genuine distributional choices—fans cannot simply hate “the outgroup” but must allocate limited emotional resources across many competitors. Second, the institutionalized nature of rivalries provides variation in relationship intensity independent of immediate interactions, allowing us to observe baseline distributional tendencies rather than purely reactive responses. Third, the stability of group boundaries and clear success metrics enable cleaner measurement than fluid political or commercial contexts where targets constantly shift. Fourth, the high emotional stakes and exclusive identity commitments characteristic of football fandom create conditions where cognitive constraints on attention allocation should be particularly visible.

We measure attention concentration using the Herfindahl-Hirschman Index, distinguishing between outgoing concentration (how communities allocate their own attention) and incoming concentration (how attention from others focuses on particular targets). Our analysis reveals systematic distributional patterns: communities consistently concentrate negative attention on fewer targets while distributing positive attention more broadly. This asymmetry persists across measurement approaches and after controlling for structural factors. Moreover, club attributes predict incoming concentration but show minimal effects on outgoing concentration, suggesting different mechanisms govern attention allocation versus attraction.

These findings demonstrate that individual-level cognitive biases produce systematic collective patterns with important implications for understanding conflict dynamics, coalition formation, and strategic positioning across competitive domains from politics to consumer markets to international relations.

## Theory

### From individual bias to collective patterns

The negativity bias—the tendency for negative information to have disproportionate psychological impact relative to equivalent positive information—is among the most robust findings in psychology [1,2]. Negative stimuli automatically capture more attention and require deeper processing than positive stimuli [3,11]. Neurological studies show stronger amygdala activation to negative than positive stimuli, with negative information receiving more elaborate encoding and stronger memory consolidation [4,12,13]. Negative emotions last longer than positive ones, and people spend more time thinking about negative events [1,5].

Research on negativity bias focuses almost entirely on individual-level processes. We know that individuals attend more to negative information, but we know much less about how these individual cognitive constraints aggregate to collective attention patterns when communities face distributional choices about emotional resource allocation.

Communities cannot effectively monitor multiple serious threats simultaneously without overwhelming processing capacity, creating pressure to concentrate negative attention on fewer targets [11]. Threat detection research shows that the cognitive system prioritizes negative stimuli for continued monitoring, while positive stimuli can be processed more peripherally [14,15]. When community members independently concentrate cognitive resources on similar negative targets due to shared cognitive limitations, their convergent focus creates collective attention concentration through social amplification processes [9].

Conversely, positive relationships require less intensive monitoring, enabling broader distribution across many targets without cognitive overload [16]. The broaden-and-build theory suggests that positive states expand attentional scope and enable more flexible cognitive processing, supporting distributed rather than concentrated attention patterns [17]. Research on emotional contagion shows that negative emotions spread more readily through social networks than positive emotions, reinforcing collective focus on threatening targets [18–20].

### Reciprocity and distribution

Reciprocity powerfully shapes which specific targets receive negative attention—communities often hate those who hate them in return [21,22]. However, reciprocity cannot fully explain distributional patterns across all available targets.

Reciprocity predicts dyadic relationships (A hates B because B hates A) but does not predict whether communities concentrate hate on few targets versus distributing it broadly across many rivals. A community facing hatred from multiple opponents could theoretically respond by reciprocating toward all of them (diffuse retaliation) or focusing retaliatory hatred on a subset (concentrated retaliation). The choice between these strategies depends on cognitive resource constraints that reciprocity alone cannot explain.

If negative emotions require more intensive processing than positive emotions due to negativity bias, then communities should concentrate their limited negative attention capacity even when multiple targets merit reciprocal hatred.

### Attention allocation in competitive contexts

Competitive social fields are structured by networks of positive and negative relationships between actors. Political parties form alliances while targeting opponents [23]. Firms collaborate in some domains while competing in others [24]. Social movements build coalitions while focusing opposition against institutional targets [6].

This distributional question matters because it determines conflict intensity and coalition possibilities. Concentrated negative attention can create intense conflicts that polarize entire fields [25], while concentrated positive attention can create dominant coalitions [26]. Conversely, diffuse attention patterns may produce manageable competition and flexible alliance formation [27].

Social network research has extensively documented the differential effects of positive and negative relationships. Negative ties influence network structure differently than positive ties [21], create distinct organizational dynamics [28], and serve different functions for group identity and cohesion [29,30]. However, this substantial literature focuses primarily on the *effects* of positive versus negative ties rather than their distributional patterns within competitive systems. Understanding how communities actually allocate positive versus negative attention across available targets represents a fundamental question that bridges individual-level cognitive processes with collective patterns of social organization.

### Alternative mechanisms

While negativity bias provides a compelling mechanism for attention concentration asymmetries, alternative mechanisms might produce different concentration patterns.

Status-based opportunity constraints might produce concentration patterns if fewer clubs represent credible high-status targets worth opposing, while positive attention faces no such constraints [31,32]. This mechanism predicts that both positive and negative attention should concentrate on the same high-status clubs, and that concentration patterns should correlate strongly across relationship types.

Uniform resource requirements suggest that communities simply lack resources to maintain intense relationships with multiple targets, forcing concentration regardless of relationship valence [33,34]. This mechanism predicts similar concentration levels for positive and negative relationships, since both types should face equivalent resource constraints.

### Hypotheses

The negativity bias mechanism generates three testable predictions about attention distribution patterns:

**Hypothesis 1 (Concentration Asymmetry):** Communities will concentrate negative attention on fewer targets than positive attention. If negative stimuli demand more intensive cognitive processing and sustained monitoring while positive stimuli can be processed more peripherally, then hate should exhibit higher concentration than love for both outgoing and incoming attention patterns.

**Hypothesis 2 (Independent Processes):** Love and hate concentration will be weakly correlated. If positive and negative attention operate through different cognitive mechanisms—with negative attention serving threat monitoring functions and positive attention serving coalition building functions—then communities should allocate them independently rather than as opposite poles of a single dimension.

**Hypothesis 3 (Agency-Structure Asymmetry):** Structural factors will predict incoming concentration but not outgoing concentration. If communities exercise agency in allocating their own attention based on internal priorities, while attracting attention based on observable characteristics, then club attributes should strongly predict how much concentrated attention clubs receive from others but have minimal effects on how clubs distribute their own attention.

## Data and methods

### Empirical context

German football provides an ideal setting for analyzing attention allocation in competitive social fields. The system includes 79 professional clubs across four competitive tiers (Bundesliga through 3. Liga), representing diverse geographic regions, historical traditions, and political contexts that generate systematic variation in affective relationship patterns.

Several institutional features enable clean identification of attention allocation mechanisms. The 50 + 1 ownership rule limits commercial control and preserves authentic community ties [35], ensuring that fan relationships reflect genuine community sentiment rather than manufactured marketing. The promotion/relegation system creates fluid competitive hierarchies where status relationships can change over time. Regional identities rooted in historical divisions between East and West Germany provide exogenous sources of group differentiation that shape contemporary attention patterns.

Most importantly, football’s institutional features enable generalization to other competitive fields. The principles governing how football fans allocate emotional attention should apply broadly to competitive contexts where communities face similar allocation problems under attention scarcity—from political partisanship to academic rivalries to organizational competition—particularly in contexts characterized by high emotional stakes, exclusive identity commitments, and repeated competitive interactions.

### Data source and network construction

We analyze data from Der Spiegel’s Fanatlas, a comprehensive 2018 survey asking 36,223 German football fans to identify their primary club and nominate up to three loved and three hated clubs (without ranking). Respondents simply listed up to three clubs in each category; no ranking or ordering was requested or provided. When a respondent’s primary club appeared among their loved nominations (rare cases), we removed the self-nomination to focus on inter-club relationships. This survey design captures both positive and negative affective ties across the full spectrum of German professional football. The survey was conducted online over two months through 62 articles on SPIEGEL ONLINE, generating nearly 60,000 reported relationships.

The survey used a convenience sampling approach and is not representative of the German football fan population. However, the large sample size (36,223 fans) and broad coverage across clubs provides sufficient data to analyze attention distribution patterns. Our findings should be interpreted as revealing patterns among German football fans who participated in this online survey, rather than nationally representative estimates of all German football supporters.

We construct club-level networks from individual nominations using inverse frequency weighting to ensure equal contribution from each fanbase regardless of survey representation. Each nomination from a fan of club A receives weight $1/n_{A}$, where $n_{A}$ is the total number of club A fans in the sample. This weighting scheme prevents large fanbases from dominating the attention networks while ensuring that smaller clubs’ attention patterns receive proportional representation.

We aggregate these weighted nominations to calculate how each club’s fanbase distributes attention across targets, enabling computation of concentration measures. The survey’s three-club nomination limit does not constrain our club-level concentration measures, since concentration is calculated based on how each club’s entire fanbase collectively distributes attention across targets. Different fans can nominate different targets, creating the full range of possible attention patterns at the aggregate level.

Of the 79 clubs in the German professional football system during the 2017−18 season, eight clubs were excluded from the analysis due to insufficient data to calculate reliable HHI measures. Specifically, clubs were excluded if they lacked sufficient fan nominations to compute at least one of the four concentration measures (outgoing love, outgoing hate, incoming love, or incoming hate concentration). The excluded clubs are: 1. FC Schweinfurt 05, Bonner SC, FC Rot-Weiss Erfurt, Rot-Weiss Oberhausen, SC Preussen Münster, SG Sonnenhof Grossaspach, Altona 93, and SV Wehen 1926 Wiesbaden. These clubs primarily represent smaller clubs or those with minimal fan representation in the survey. Importantly, these excluded clubs still contributed to the attention flows when computing concentration measures for the 71 clubs retained in the analysis—their fans’ nominations were included in calculating how attention focuses on other clubs, ensuring that incoming concentration measures capture the full network structure. The exclusion affects only the calculation of these specific clubs’ own concentration patterns, not their role as targets of attention from other clubs’ fanbases.

### Ethics statement

This study uses anonymized, publicly available survey data collected by Der Spiegel. No human subjects were directly recruited, and no ethics approval was required for this secondary data analysis.

### Analytical strategy and measures

We measure attention concentration using the Herfindahl-Hirschman Index (HHI):


$$\begin{array}{c} HHI_{i}=\sum_{j}s_{ij}^{2} \end{array}$$ (1)

where $s_{ij}$ represents the share of club *i*’s total attention directed toward target *j*. HHI values range from 1/*n* to 1, with higher values indicating more concentrated attention distributions.

We employ HHI because negativity bias theory specifically predicts effects in top-target attention—cognitive systems should concentrate processing resources on the most threatening stimuli [11]. HHI is particularly sensitive to concentration in the right tail of the distribution, making it well-suited to detect this mechanism. We validate findings using Gini coefficients, Shannon entropy, and top-3 concentration measures, which weight the full distribution differently, ensuring results are not artifacts of HHI’s specific properties.

We calculate two types of concentration measures for each club: (1) Outgoing concentration: How club *i*’s fanbase distributes its own attention across targets, and (2) Incoming concentration: How attention from all other clubs’ fanbases focuses on club *i*. This distinction proves crucial for understanding different mechanisms governing attention allocation versus attraction.

Since HHI values are bounded between 0 and 1, we employ beta regression models to properly account for the distributional properties of our concentration measures [36]. Beta regression is specifically designed for response variables that take values in the (0,1) interval and provides more appropriate inference than OLS regression for bounded dependent variables.

All continuous variables are standardized (mean-centered and scaled by standard deviation) before entering the regression models, allowing direct comparison of effect sizes across variables. League tier remains as a categorical variable with Bundesliga (1st division) as the reference category.

The analysis is not intended as a causal identification strategy. Its inferential advantage lies instead in observing the distribution of relational attention across the full set of potential targets. Comparing positive and negative concentration, distinguishing outgoing from incoming concentration, and assessing robustness across alternative concentration measures allows us to test distributional implications that cannot be recovered from individual dyadic relationships alone.

### Control variables

We incorporate multiple control variables reflecting factors that might influence attention allocation patterns: Structural factors include league tier (1st through 4th division), stadium capacity (logged), club age (years since founding), and market value (Transfermarkt estimates of player values in 2018, in millions, logged), capturing competitive status, resource availability, and institutional prominence. Geographic factors include regional GDP per capita and unemployment rates, reflecting local economic conditions that may influence fan behavior and attention allocation patterns. Historical factors include a binary East/West variable identifying clubs from former East Germany, capturing legacy effects of German division on contemporary attention allocation patterns. Political factors include 2017 AfD (Alternative for Germany, a right-wing populist party) vote share in each club’s region, capturing contemporary political cleavages that may align with or cross-cut football attention allocation patterns. Performance factors include the club’s final ranking in the 2017−18 season and the average ranking over the previous 10 seasons (where lower values indicate better performance), capturing both recent success and historical competitive standing. Table 1 presents descriptive statistics for all variables in the analysis sample of 71 clubs.

**Table 1:** Descriptive statistics.

Variable	Mean	SD	Min	Max
**Concentration Measures (HHI)**				
Love Concentration (Outgoing)	0.25	0.18	0.00	0.76
Hate Concentration (Outgoing)	0.37	0.16	0.00	0.73
Love Concentration (Incoming)	0.30	0.21	0.02	0.96
Hate Concentration (Incoming)	0.39	0.22	0.04	0.97
**Structural Factors**				
Stadium Capacity (log)	10.03	0.64	8.41	11.31
Club Age (years)	96.06	34.57	8.00	158.00
Market Value (log)	2.41	1.70	−0.01	6.45
Members (log)	9.17	1.66	6.43	12.90
**Geographic Factors**				
GDP per Capita	40804.23	9195.22	27300.00	64000.00
Unemployment Rate	5.06	1.73	2.05	9.45
**Historical/Political**				
AfD Vote Share	12.94	5.64	5.57	30.46
**Performance (lower = better)**				
Ranking (last season; overall)	34.77	18.75	1.00	56.00
Avg. Ranking (prev. 10 seasons; overall)	37.55	16.13	6.90	56.00

**Categorical variables:**

League Tier: Bundesliga (n = 18), 2nd Division (n = 16), 3rd Division (n = 16), Regional (n = 21).

East Germany: Yes (n=15), No (n=56).

Total N=71 clubs (8 clubs excluded due to insufficient data).

## Results

### Concentrated hate, diffuse love

**Fig 1:**
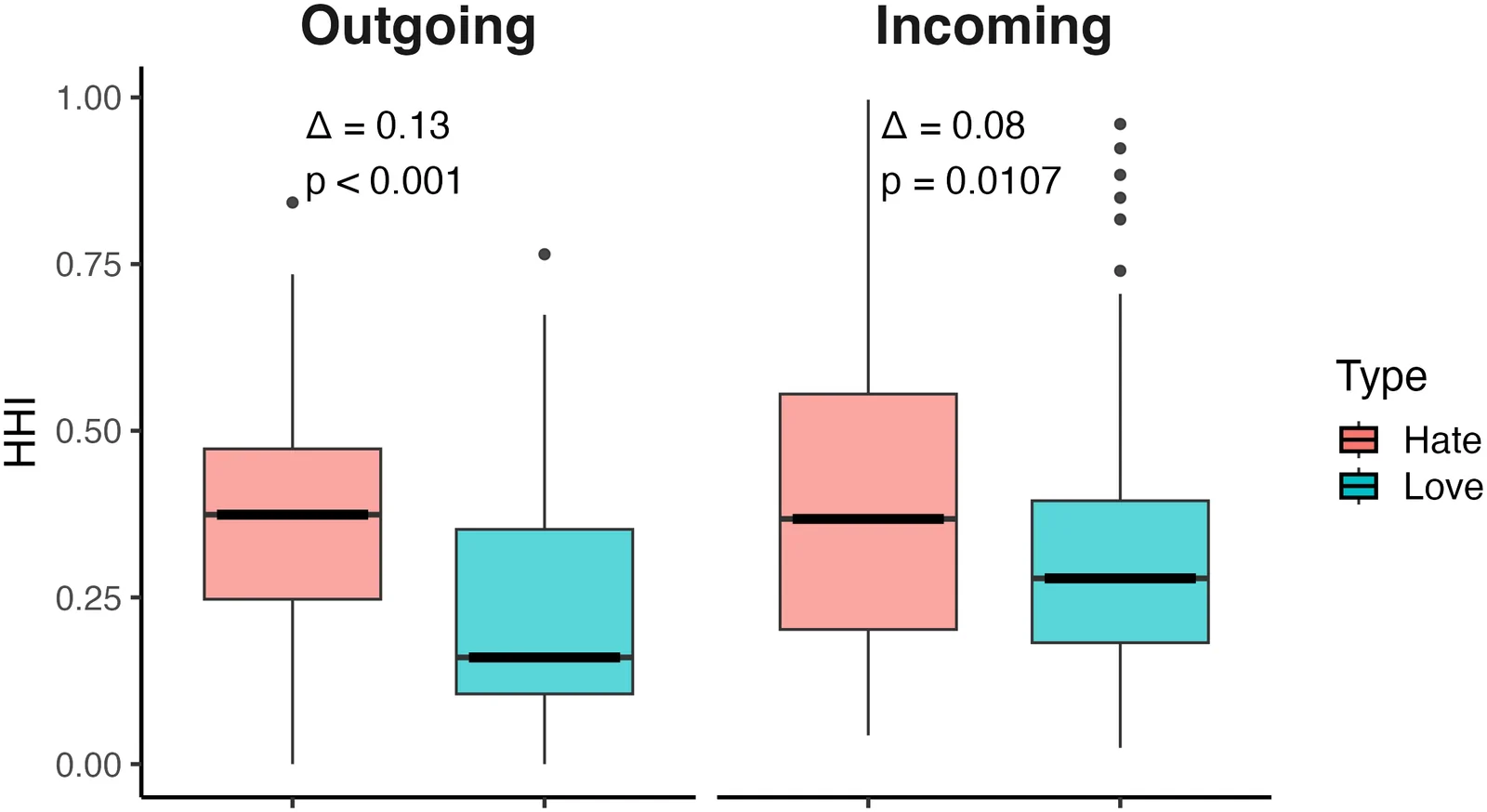
Attention concentration patterns across German football. Box plots showing the distribution of love and hate concentration for both outgoing and incoming ties. Hate concentration (red) significantly exceeds love concentration (blue) in both directions ($\Delta_{outgoing}=0.13$, *p* < 0.001; $\Delta_{incoming}=0.08$, *p* = 0.011).

Fig 1 reveals the core distributional asymmetry characterizing attention allocation in competitive fields. Communities systematically concentrate negative attention on fewer targets while distributing positive attention more broadly. For outgoing ties, hate concentration (*M* = 0.36, *SD* = 0.18) significantly exceeds love concentration (*M* = 0.23, *SD* = 0.18), with a substantial difference of Δ=0.13 (*p* < 0.001). For incoming ties, hate concentration (*M* = 0.41, *SD* = 0.24) similarly exceeds love concentration (*M* = 0.33, *SD* = 0.22), with a difference of Δ=0.08 (*p* = 0.011). This supports **H1**: negative attention is more concentrated, positive attention more diffuse.

To provide practical interpretation of these concentration differences, consider that the mean hate concentration of 0.36 indicates that the average club’s fanbase directs roughly one-third of their total hate toward a single rival, while the remaining two-thirds is distributed among other targets. In contrast, the mean love concentration of 0.23 indicates that positive attention is spread more evenly, with less than one-quarter directed toward any single club. This difference represents a meaningful divergence in attention allocation strategies, where negative emotions focus intensely on primary rivals while positive emotions distribute across multiple clubs.

### Independence of positive and negative patterns

**Fig 2:**
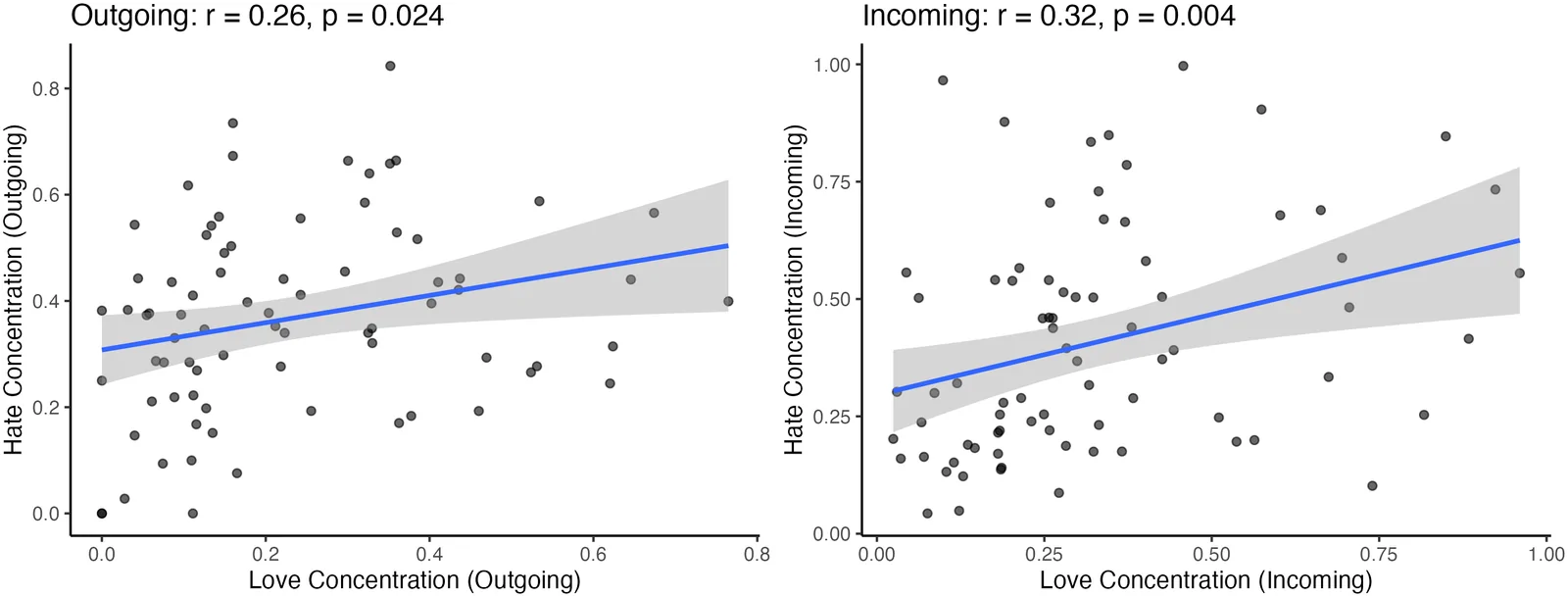
Correlation of hate and love concentrations. Scatter plots showing the relationship between love and hate concentration for outgoing (left) and incoming (right) ties. Weak correlations (*r* = 0.26–0.32) indicate largely independent processes, with substantial scatter demonstrating that communities allocate positive and negative attention independently.

Correlations between love and hate concentration are weak to moderate, supporting **H2**. For outgoing concentration, *r* = 0.26 (*p* = 0.024); for incoming concentration, *r* = 0.32 (*p* = 0.004). These modest correlations indicate that positive and negative attention operate through largely independent processes rather than opposite poles of a single dimension. Communities that concentrate their hate do not systematically concentrate or diffuse their love, suggesting distinct cognitive and strategic mechanisms govern each type of attention allocation. Fig 2 visualizes these relationships, revealing substantial scatter around the regression lines despite statistically significant correlations. The weak to moderate associations demonstrate that while some clubs may coordinate their love and hate concentration strategies, the majority allocate positive and negative attention through independent decision-making processes.

### Agency versus structure

Table 2 strongly supports **H3**, revealing fundamentally different mechanisms governing how communities allocate attention versus how they attract it. Club attributes show minimal effects on outgoing concentration patterns, with modest explanatory power for both love (Pseudo *R*^2^ = 0.35) and hate (Pseudo *R*^2^ = 0.14) outgoing concentration. For outgoing attention, only second division status significantly predicts love concentration (β=1.10, *p* < 0.01), while GDP per capita shows a significant positive effect on hate concentration (β=0.38, *p* < 0.01). The generally weak effects indicate that communities exercise substantial agency in distributing their own attention largely independent of their club’s observable characteristics.

**Table 2:** Beta regression results for attention concentration.

	Outgoing		Incoming	
	Love	Hate	Love	Hate
**Structural Factors**				
Stadium capacity	0.22 (0.20)	–0.21 (0.22)	0.08 (0.19)	–0.64^***^ (0.19)
Club age	–0.06 (0.12)	0.03 (0.13)	–0.04 (0.11)	–0.28^**^ (0.10)
Market value (log)	–0.42 (0.34)	–0.49 (0.37)	–0.76^*^ (0.33	–0.57^†^ (0.31)
Members (log)	0.29 (0.19)	0.37^†^ (0.21)	–0.27 (0.19)	0.20 (0.17)
**Geographic Factors**				
GDP per capita	0.04 (0.13)	0.33^*^ (0.13)	–0.07 (0.12)	–0.21^†^ (0.11)
Unemployment rate	–0.14 (0.14)	0.08 (0.15)	–0.01 (0.14)	–0.33^*^ (0.13)
**Historical/Political**				
East Germany	0.23 (0.56)	0.42 (0.61)	–1.87^***^ (0.54)	0.48 (0.51)
AfD vote share	0.23 (0.18)	0.17 (0.19)	0.55^**^ (0.17)	–0.28^†^ (0.16)
**League Tier (ref: Bundesliga)**				
2nd division	1.10^**^ (0.38)	0.40 (0.41)	–0.00 (0.37)	0.06 (0.35)
3rd division	0.13 (0.52)	–0.38 (0.56)	0.15 (0.49)	–0.26 (0.47)
Regional	0.63 (0.61)	0.90 (0.66)	0.75 (0.58)	0.47 (0.56)
**Performance (lower = better)**				
Ranking (last season)	–0.01 (0.01)	–0.01 (0.02)	–0.04^**^ (0.01)	–0.02^†^ (0.01)
Avg. ranking (prev. 10 seasons)	–0.00 (0.01)	–0.03^*^ (0.02)	0.00 (0.01)	–0.01 (0.01)
Pseudo *R*^2^	0.36	0.15	0.51	0.53
Log-likelihood	49.89	43.34	43.32	38.64
N	71	71	71	71

Table notes: Coefficients from beta regression models with logit link shown with standard errors in parentheses. ^†^ *p* < 0.10, ^*^ *p* < 0.05, ^**^ *p* < 0.01, ^***^ *p* < 0.001. Eight clubs were excluded due to insufficient data for HHI calculation.

In sharp contrast, attracting concentrated attention depends heavily on observable characteristics and structural position. The models predicting incoming concentration show strong explanatory power for both love (Pseudo *R*^2^ = 0.47) and hate (Pseudo *R*^2^ = 0.49). East German clubs receive significantly lower incoming love concentration (β=−1.80, *p* < 0.001), indicating broader-based affection that likely reflects historical solidarity. For incoming hate concentration, several structural factors matter: larger stadium capacity (β=−0.65, *p* < 0.001) and older clubs (β=−0.25, *p* < 0.05) receive more diffuse hate, while clubs in higher unemployment regions attract less concentrated hate (β=−0.36, *p* < 0.01). Political context also matters for incoming love: regions with higher AfD support generate more concentrated love (β=0.56, *p* < 0.001), suggesting that political polarization aligns with attention concentration patterns.

Performance variables reveal additional nuances in attention attraction. Clubs with better recent performance (lower ranking values) attract more concentrated incoming love (β=−0.04, *p* < 0.01), suggesting that successful clubs become focal points for positive attention from specific fanbases, possibly their own supporters or affiliated communities. Similarly, better recent performance marginally predicts more concentrated incoming hate (β=−0.02, *p* < 0.10), indicating that success can make clubs targets of focused animosity. Historical performance over the previous decade shows a significant effect on outgoing hate concentration (β=−0.03, *p* < 0.05), suggesting that clubs with histories of greater success allocate their own negative attention more diffusely, perhaps because sustained competitive standing creates multiple rivalries that distribute hate across several opponents rather than focusing intensely on a single rival.

### Robustness checks

**Alternative concentration measures.** The Gini coefficient confirms our primary findings, with outgoing hate Gini (0.672) significantly exceeding outgoing love Gini (0.578), *p* < 0.001, while incoming hate and love Gini are similar (0.732 vs 0.725). Shannon entropy analysis (where lower values indicate higher concentration) similarly demonstrates that hate attention exhibits lower entropy than love attention for both outgoing (1.216 vs 1.689, *p* < 0.001) and incoming ties (1.221 vs 1.578, *p* < 0.001). Top-3 concentration measures (proportion of attention directed toward the top 3 targets) further confirm the concentration asymmetry: outgoing hate concentrates 87.4% of attention in the top 3 targets compared to 74.2% for love (*p* < 0.001), with similar patterns for incoming ties (85.8% vs 78.1%, *p* < 0.001).

## Discussion

### Summary of findings

Taken together, the results support all three hypotheses: negative attention is more concentrated than positive attention (H1), love and hate concentration are only weakly associated (H2), and observable structural characteristics explain incoming concentration substantially better than outgoing concentration (H3).

Our analysis provides strong evidence that individual-level negativity bias scales up to produce systematic collective patterns in emotional attention allocation. Communities consistently concentrate negative emotions on fewer targets while distributing positive emotions more broadly—an asymmetry that persists across multiple measurement approaches, different subgroups, and after controlling for structural factors. The weak correlations between love and hate concentration demonstrate that positive and negative attention operate through largely independent processes. Communities exercise substantial agency in allocating their own attention based on internal priorities, while attracting attention based on observable characteristics and structural position—revealing fundamentally asymmetric mechanisms.

### Theoretical contributions

These findings advance understanding of how psychological mechanisms operating at the individual level produce collective patterns of social organization. The negativity bias—a robust individual-level phenomenon—generates systematic asymmetries in collective attention distribution when communities face allocation constraints. The agency-structure asymmetry we identify challenges prevailing assumptions about relationship formation. Most network formation models assume symmetric processes where similar mechanisms govern both tie initiation and reception [37,38], but attention networks appear fundamentally asymmetric. Actors can control their outgoing attention strategies but have limited influence over how others attend to them.

The finding that East German clubs receive significantly more diffuse love concentration (β=−1.80, *p* < 0.001) illustrates how historical experiences of solidarity can create lasting patterns of broader-based affection that persist decades after reunification. This demonstrates how structural legacies interact with attention allocation processes, creating systematic variation in distributional patterns across different social contexts.

### Ruling out alternative explanations

Our empirical findings enable evaluation of alternative mechanisms. Status-based opportunity constraints cannot explain the weak correlations between love and hate concentration—if both focused on the same high-status targets, we should observe stronger associations. The substantial scatter in Fig 2 demonstrates that communities do not simply coordinate their positive and negative attention on similar high-status clubs.

Uniform resource requirements similarly cannot account for the systematic concentration asymmetry. If cognitive constraints affected both relationship types equally, we should see similar concentration levels for positive and negative attention rather than the consistent differences documented in Fig 1.

The agency-structure asymmetry further contradicts explanations based solely on structural positioning. If attention allocation were determined entirely by structural factors, we should observe similar explanatory power for both outgoing and incoming concentration. Instead, Table 2 reveals that communities exercise choice in their attention allocation strategies (low *R*^2^ for outgoing models) while their structural position largely determines how others attend to them (high *R*^2^ for incoming models).

### Broader implications

The attention concentration asymmetries we document extend beyond football to multiple domains where communities manage scarce emotional resources under competitive conditions.

#### Political polarization.

Contemporary political discourse exhibits similar patterns, with negative partisan affect increasingly concentrating on specific opposing figures [8, 39] while positive political identification distributes across multiple candidates and issues. Our findings suggest this asymmetry may reflect fundamental cognitive constraints rather than strategic manipulation alone. Interventions targeting attention redistribution—through institutional design or media regulation—may prove more effective than traditional approaches focusing solely on promoting positive contact between groups.

#### Consumer behavior.

Brand hatred concentrates intensely on particular competitors while brand appreciation spreads across numerous alternatives [7]. Marketing strategies assuming symmetry between positive and negative brand relationships may misunderstand how consumers actually allocate emotional attention. Understanding concentration asymmetries could inform more effective brand positioning and competitive strategy.

#### Social media dynamics.

Online platforms compete for user engagement in crowded attention markets where negative content demonstrably spreads more rapidly than positive content [9,10,40,41]. Understanding why negative attention naturally concentrates may inform platform design and content moderation strategies. Algorithms optimizing for engagement may inadvertently amplify natural concentration asymmetries, creating feedback loops that intensify polarization.

#### Conflict resolution.

Interventions promoting positive intergroup contact may face inherent limitations if positive attention is already naturally diffuse while negative attention remains concentrated. Successful prejudice reduction may require specifically addressing concentrated negative attention rather than assuming positive contact can symmetrically counteract it. The finding that clubs in higher unemployment regions attract less concentrated hate (β=−0.36, *p* < 0.01) suggests that economic context may influence negative attention patterns, though the specific mechanisms remain unclear. This negative coefficient indicates that hate directed toward clubs in economically disadvantaged regions comes from more distributed sources rather than focusing from specific rivals. Whether this reflects broader economic grievances, reduced rivalry intensity, or other factors requires further investigation.

The particularly strong effect showing that regions with higher AfD support generate more concentrated love (β=0.56, *p* < 0.001) provides direct evidence that contemporary political polarization aligns with emotional attention patterns, suggesting these mechanisms operate across different domains of social competition.

### Boundary conditions and generalizability

Several contextual features of football fandom warrant consideration when generalizing these findings to other competitive domains.

Football involves particularly exclusive and emotionally intense identity commitments. Fans typically support one club exclusively, creating clear boundaries that amplify the salience of rivalry relationships. Contexts involving less exclusive identities (e.g., professional networks where individuals maintain ties with multiple organizations) may exhibit different concentration patterns.

The zero-sum nature of sporting competition—where one club’s victory necessarily means another’s defeat—creates inherent conflicts of interest that may strengthen concentration asymmetries. Contexts allowing for positive-sum relationships or shifting alliances might show weaker effects.

The institutionalized structure of German football, with clear hierarchies, repeated interactions, and stable club identities, provides ideal conditions for observing baseline attention allocation patterns. More fluid competitive environments with frequently changing actors and relationship structures might exhibit different dynamics.

However, multiple features suggest these patterns should generalize to other high-stakes competitive contexts: (1) the cognitive constraints underlying negativity bias operate universally across cultures and contexts [1], (2) attention scarcity affects all communities regardless of domain, and (3) similar concentration asymmetries appear in political partisanship, consumer brand relationships, and online discourse, suggesting common underlying mechanisms.

Future research should test whether concentration asymmetries persist in contexts varying in identity exclusivity, competitive structure, and institutional stability to establish precise boundary conditions. While football fandom provides a high-stakes competitive context, future research should examine whether similar attention concentration asymmetries appear in less exclusive domains.

### Sample characteristics and external validity

The convenience sample used in this study, while large (N = 36,223 fans), may not perfectly represent the full German football fan population. The online recruitment through Der Spiegel articles likely attracted more engaged, internet-connected fans who actively follow football coverage. This may affect the generalizability of our findings in several ways.

First, highly engaged fans may exhibit stronger emotional attachments and clearer rivalry patterns than casual supporters, potentially amplifying the concentration asymmetries we observe. If casual fans distribute their attention more evenly across both positive and negative targets, our estimates may represent upper bounds on concentration differences in the broader population.

Second, the online nature of the survey may underrepresent older fans, fans from rural areas with lower internet penetration, and fans from lower socioeconomic backgrounds who consume football coverage through different media channels. To the extent that attention allocation patterns vary across these demographic groups, our findings may not fully capture population-level heterogeneity.

Third, self-selection into a survey about club preferences likely attracts fans with stronger opinions and clearer loyalty/rivalry structures. Fans who lack strong feelings about multiple clubs may be less motivated to participate, potentially leading to overestimation of both positive and negative attention concentration.

However, several factors suggest that the core theoretical findings—that negative attention concentrates more than positive attention—likely generalize beyond this specific sample. The negativity bias operates as a fundamental cognitive mechanism across populations [1], and the substantial sample size provides statistical power to detect systematic patterns despite potential sampling biases. Moreover, the theoretical mechanisms we identify (cognitive constraints on threat monitoring, independent processing of positive versus negative affect) should operate similarly across fan engagement levels, even if the absolute magnitude of concentration differs.

Future research should replicate these findings using representative sampling methods, alternative fan populations (e.g., other sports leagues, other countries), and behavioral measures of attention allocation (e.g., social media engagement, attendance patterns) to establish the robustness and boundary conditions of these patterns. Longitudinal designs tracking the same fans over time would enable stronger causal inference about how attention concentration patterns evolve and respond to changing competitive contexts.

### Limitations and future directions

Several limitations qualify our findings and suggest important directions for future research.

Our analysis includes 71 of the 79 clubs in the dataset, as eight clubs had to be excluded due to insufficient nomination data for reliable HHI calculation. These clubs primarily represent smaller or regional clubs with limited fan representation in the survey. This exclusion could potentially bias results toward clubs with larger, more active fanbases, though the systematic patterns we observe suggest our findings reflect general principles rather than artifacts of sample composition.

The cross-sectional design limits causal claims about attention allocation processes and their consequences. Longitudinal research tracking concentration changes following major events such as promotion/relegation decisions, ownership changes, or political campaigns would enable stronger causal inference about whether attention concentration patterns cause competitive outcomes or emerge as consequences of structural positioning.

Survey-based preference measures may not fully capture actual attention allocation behavior, creating potential gaps between stated and revealed preferences. Future research should triangulate findings using behavioral measures such as social media engagement patterns, resource allocation decisions, or temporal attention tracking.

Our analysis focuses on dyadic attention relationships but cannot capture higher-order network effects that may be crucial for understanding system-level outcomes. Do communities with concentrated hate trigger concentration responses in their targets, creating escalating conflict spirals? Network-based studies could reveal emergent dynamics that arise from individual attention allocation decisions but cannot be predicted from dyadic patterns alone.

## Conclusion

Communities systematically concentrate negative emotions on fewer targets while distributing positive emotions more broadly. This asymmetry persists across multiple measurement approaches and after controlling for structural factors, demonstrating how individual-level cognitive constraints scale up to shape collective emotional structures.

Our central theoretical contribution is an agency-structure asymmetry in network formation: communities exercise substantial agency in allocating their own attention, with structural factors explaining little variance in outgoing concentration, while attracting attention depends heavily on observable characteristics, with much higher explanatory power for incoming concentration. This challenges prevailing assumptions that symmetric mechanisms govern both tie initiation and reception in social networks.

These findings have practical implications for understanding political polarization, consumer competition, and online discourse, where systematic concentration of negative attention shapes conflict dynamics and coalition possibilities in ways that interventions focused solely on promoting positive contact may not adequately address.
